# Bridging the gap in ageing: Translating policies into practice in Malaysian Primary Care

**DOI:** 10.1186/1447-056X-10-2

**Published:** 2011-03-08

**Authors:** Krishnapillai S Ambigga, Anis Safura Ramli, Ariaratnam Suthahar, Norlaili Tauhid, Lyn Clearihan, Colette Browning

**Affiliations:** 1 Discipline of Primary Care Medicine, Faculty of Medicine, Universiti Teknologi MARA (UiTM), 40450 Shah Alam, Selangor, Malaysia; 2 Department of General Practice, School of Primary Health Care, Monash University, Notting Hill, 3168 Victoria, Australia; 3 Healthy Ageing Research Unit, School of Primary Health Care, Monash University, Notting Hill, 3168 Victoria, Australia

## Abstract

Population ageing is poised to become a major challenge to the health system as Malaysia progresses to becoming a developed nation by 2020. This article aims to review the various ageing policy frameworks available globally; compare aged care policies and health services in Malaysia with Australia; and discuss various issues and challenges in translating these policies into practice in the Malaysian primary care system. Fundamental solutions identified to bridge the gap include restructuring of the health care system, development of comprehensive benefit packages for older people under the national health financing scheme, training of the primary care workforce, effective use of electronic medical records and clinical guidelines; and empowering older people and their caregivers with knowledge, skills and positive attitudes to ageing and self care. Ultimately, family medicine specialists must become the agents for change to lead multidisciplinary teams and work with various agencies to ensure that better coordination, continuity and quality of care are eventually delivered to older patients across time and settings.

## Introduction

Population ageing remains a global phenomenon in this new millennium and is poised to become a major issue in developing countries. Malaysia, an upper middle income country, with a population of 28 million is no exception [1]. Its ageing population of 60 years and above is rising steadily from 5.7% in 1990 to 6.3% in 2000 and is expected to be 9.8% in 2020 [2]. Like other countries, population ageing in Malaysia is a result of declining fertility, falling mortality rates and improvements in the health system. Effective prevention of infectious diseases and better nutrition has resulted in more people surviving into old age [3,4]. Life expectancy among Malaysians has also risen to 71.7 years for men and 76.5 years for women in 2007 [5]. Despite the improvement in life expectancy; poverty, lack of education and poor social support tend to influence the well being of its older population.

In comparison to Australia, a developed country with 22 million population, 13.3% of its populace are aged 65 years and over [6]. Life expectancy for Australian males is 79 years and for females is 84 years [6]. The older population in Australia are healthier and better educated than their Malaysian counterparts.

Whilst the definitions of chronological age for the older people in Malaysia and Australia are different, comparison can still be made because chronological age is not a precise marker for the biological changes which accompany ageing. The chronological age of 60 years and above seems young in the developed world, but for a developing country such as Malaysia where gains in life expectancy have not yet matched the developed world, this definition is more appropriate [7]. Chronological age has little or no importance in the meaning of old age in many parts of the developing world [8]. Other socially constructed meanings of age may be more significant such as the roles assigned to older people. In some cases, it is the loss of roles accompanying physical decline which is significant in defining old age.

A Malaysian burden of disease study conducted in 2004 using disability adjusted life years (DALY) showed that the five leading causes of disease burden were coronary heart disease, mental illnesses, cerebrovascular disease, road traffic injuries and cancer [9]. This trend is similar to that in Australia as the five leading causes of disease burden using DALY in 2003 were coronary heart disease, anxiety and depression, type 2 diabetes mellitus, cerebrovascular disease and dementia [10]. Hence, Malaysia is at an epidemiological transition where non-communicable diseases are now dominating its burden at a par with those of developed countries. This pattern of disease is especially predominant among the older population in both countries [10,11].

As Malaysia progresses in its path to becoming a developed nation by 2020, population ageing is inevitable and this will generate new challenges in terms of health and social services [11]. In view of this phenomenon, this article aims to review the various ageing policy frameworks available globally; dissect various issues, policies and health services pertaining to the older population in Malaysia; discuss the challenges of translating these policies into practice in the Malaysian primary care system; and consider fundamental solutions needed to address improving the health of older people in Malaysia.

### Ageing policy frameworks available globally

Issues involving the ageing society in each country are unique, resulting in the adoption of various frameworks for ageing throughout the world. A number of these are summarised in Table 1.

**Table 1:** Various ageing policy frameworks available globally

Policy frameworks	Definitions	Countries Adopting
**Active Ageing (WHO)**	Continuing participation in social, economic, cultural, spiritual and civic affairs by older persons and not just being physically active or mere participation in the labour force [12].	United Kingdom and Europe

**Active Ageing (Adapted)**	Optimizing opportunities for physical, social, mental well being throughout life, in order to extend healthy life expectancy, productivity and good quality of life as people age [13].	Malaysia

**Healthy Ageing**	All Australians have the opportunity to maximise their physical, social and mental health throughout life. Population health strategies promote and support healthy ageing across the lifespan. Information, research and health care infrastructure is available to support the healthy ageing of the Australian population [14].	Australia

**Successful Ageing**	Multiple dimensions of functioning and wellness are measured and these include cognitive and affective status, overall physical health, social functionally and life engagement including life satisfaction. These will form the salient determinants of successful ageing [15].	Singapore

**Healthy Ageing**	A lifelong process of optimising opportunities for improving and preserving health and physical, social and mental wellness, independence, quality of life and enhancing successful life-course transitions [16].	Canada

**Positive Ageing**	Shine a positive light on ageing and older persons by recognizing their potential skills and ability rather than their age [17].	New Zealand

These frameworks cover various domains which include longevity, physical health, activities of daily living, autonomy, psychological well being, social relationships, work participation, financial security, housing, transport, safety, leisure activities, quality of life, age discrimination and attitudes [12-17]. These frameworks and their attendant policies help guide the development of relevant programmes, facilities and services around them.

These various policy frameworks for the different countries are designed based on the rights, needs, preferences and capacities of older people in each country hence different domains have been chosen by the various countries. Since there are dramatic variations in health status, participation and levels of independence among older people of the same age in different countries, decision-makers need to take this into account when designing policies and programmes for their older populations.

### Comparing Aged Care Policies of Malaysia with Australia

In most Asian countries, co-residing with an older relative and providing aged care is part of the cultural tradition. Policy makers in Asia aim at maintaining these cultural norms and values rather than developing potentially expensive formal aged care programs and facilities. These policies focus on the older person remaining integrated in society and being included in the formulation and implementation of policies that affect their wellbeing [3]. In Malaysia, a multi-sectoral and multidisciplinary approach was required to set up these policies and they emphasized the sharing of responsibility between government, private sectors, non-government organisations, community and the older people themselves in order to meet their needs [18].

There have been a number of national policies put in place for older people in Malaysia. The first policy was the National Social Welfare Policy (1990).This policy addressed the need for the care of older persons by families and communities [18].This was later strengthened with the National Policy for the Elderly (1995) aimed at "creating a society of elderly people who are contented and possess a high sense of self worth and dignity, by optimising their self potential and ensuring that they enjoy every opportunity as well as care and protection from members of their family, society and nation" [7]. The National Council of Senior Citizen's Organization Malaysia (NACSOM), the Gerontological Association of Malaysia (GEM) and others provide the impetus in developing these policies and services for older people in Malaysia [4].

In contrast, ageing policy in Australia reflects different cultural norms and focuses on consolidating and progressing reforms to ensure choice and access to quality aged care services while relying on the role of informal support [19]. In Australia, the focus is on ageing in place through community support and services. Nevertheless this is changing towards person-centred approaches that promote independence.

Societal pressures and expectations are changing in Malaysia with an increasing number of older people residing in the cities following the rural-urban migration; the changing pattern of families into nuclear types; and the changing role of women from caregivers to wage earners to support their families. Therefore, it is timely for Malaysia to revise its policies and be prepared to invest more in quality aged care services in response to these changes in cultural norms and values.

### Health issues involving the ageing society in Malaysia

Health issues pertaining to ageing are unique for each society. In order to gain a deeper understanding of the health issues in the Malaysian ageing population, we conducted a comprehensive literature review of the health and medical research involving Malaysian elders using electronic databases including Medline, Google Scholar, PubMed, Scopus and Web of Science. Based on the published literature, ageing health issues in Malaysia can be summarised into 3 main domains - physical health, psychosocial health and nutritional problems.

According to the Third National Health and Morbidity Survey (NHMS III), chronic illnesses were reported to be most prevalent (48.8%) amongst the 60 and above age group [20]. Malaysian elders are commonly affected by multiple chronic non-communicable diseases such as hypertension, type 2 diabetes, coronary heart disease and stroke as evidenced by the findings in the literature[20-22].

This is hardly surprising as the prevalence of cardiovascular risk factors among the Malaysian elderly are increasing. In a large population based study, the prevalence of obesity among the elderly were 8.8% in males and 13.2% in females [23]. Where else, the prevalence of abdominal obesity in the 60-69 years age group was 23.2%, followed by 19% in the 70-79 years group and 14.9% in the 80 years and above group [24]. However, older persons who reside in publicly funded shelter homes were found to be at risk of being undernourished and underweight[25,26].

Visual impairment and blindness were noted to be high in the older population and there is a need to involve ophthalmic and optometric services as part of comprehensive medical care in the community [27].There is also an increasing pattern of orthopaedic diseases in the ageing population [28] and functional impairment was found to be common among older people in the community [29]. Based on the NHMS III report, the greatest impact of functional independence in the elderly is on mobility, self care, housework and access to public places [20]. The survey also reported a higher prevalence of chronic pain in the elderly which interfered with their daily activities [20].

Another study also found that erectile dysfunction is prevalent and this usually co-exists with type 2 diabetes and depression [30]. Urinary incontinence is an important and common problem and can lead to poor quality of life without timely management [31].

Psychological health problems are also prevalent among older people in Malaysia [32]. A community survey conducted in an urban area found that the prevalence of depression in the elderly was 6.3% [33]. The prevalence of depression among the elders attending a health clinic was reported to be even higher at 18% [34]. In this age group, depression often co-exists with other chronic illnesses [35]. This condition is common in primary care and therefore providers should play a key role in the detection and management [36].

The other non-health factors which should be addressed in Malaysia includes work, retirement and income, housing and institutionalisation, family and the community, leisure and personal characteristics which can influence the quality of life of this vulnerable group [3]. These however, are beyond the scope of this paper.

In summary, published evidence has repeatedly highlighted that older Malaysian population suffers from multiple and complex health needs which require holistic and comprehensive long term care in the community. Despite this fact, Malaysia struggles to translate its policies into practice. A closer scrutiny of how the Malaysian health system is responding to the needs of its ageing population is therefore pivotal.

### Caring for older people in the health care system: current situation and challenges

Malaysian health care services are provided by both the public and private sectors. The establishment of geriatric secondary care services has occurred since the mid 1990's and was an important milestone in the care of the older patients [7]. Currently, there are 3 public hospitals and 1 private hospital offering care for older patients [7]. Rehabilitation centres are also being established throughout Malaysia. However, there are only 11 geriatricians available throughout the country and the majority are based in Kuala Lumpur [37]. There is a constant challenge to recruit and retain key medical and allied health personnel in the public health sector, as many choose to leave for the private sectors. This further prevents expansion of secondary care support services into the community.

The expansion of services for older people in Malaysian primary care is even slower despite the need for long-term management of this vulnerable group in the community. Older persons in Malaysia visit the public and private primary care services on an average of 6 visits per year [38]. In view of this, primary care providers remain a popular first contact in the community. However, Malaysian primary health care system, be it the public or private sectors, remain oriented towards the care of acute, episodic illnesses [21]. The health system remains passive with older patient seeking treatment only when symptoms develop, leaving the need of older people with complex and multiple chronic conditions unfulfilled [3]. Although preventive and other specialised programmes for the older people in the community were planned, their implementation in public primary care clinics is sporadic. The main impediment remains the shortage of a trained primary care workforce. Although there are approximately 10,000 primary care doctors currently manning the frontline of its public and private primary health care systems, only around 400 are qualified family medicine specialists [39]. Out of the 400, only 6 have completed subspecialist training in community geriatrics [39]. Shortage of trained family medicine specialists and allied health personnel also hampers the deployment of multidisciplinary primary care teams much needed to care for long term and complex health problems in the community.

Fragmentation of primary health care services into public and private sectors remains the greatest challenge in providing comprehensive, coordinated and continuous care, especially for those with multiple chronic conditions and complex health care needs [21]. The absence of a universal funding mechanism results in unequitable access to health care. The public primary care sector is often over-burdened and over-subsidised to provide care, especially to the vulnerable groups such as older patients. In the private sector, health care costs for older patients with multiple and complex conditions are often too expensive for them to bear the out-of-pocket payment. The current fee-for-service payment to the private general practitioners (GPs) by the private health insurance companies and employers, results in limitation of care for those with multiple and complex health care needs who often require longer consultations and home visits. Often, comprehensive care packages such as these are not covered.

In comparison, the Australian health care system is also complex. There are many types of service providers and a variety of funding and regulatory mechanisms in its system. Services are provided by medical practitioners, allied health professionals and other government and non-government agencies [40]. Australia's health services are grouped into five broad categories: public health services; primary care and community health services; hospitals; specialised health services; and goods such as medicine [41]. Funding is provided by the Australian Government through the national health insurance scheme (Medicare), state and territory governments, private health insurers, individual Australians (out-of pocket payments) and a range of other sources [40].

Primary care and community health services are usually the first health services visited by a patient with a health concern in Australia. These services include GPs, private dentists, pharmacists, physiotherapists and various other practitioners. The average number of visits for older Australians to their GP was 8.6 per person per year [40]. Annual health checks for patients above 75 years old in primary care are remunerated by Medicare. There are also community based aged care assessment teams (ACAT) to assess the elderly who may need special assistance [40]. The approval of these teams is a prerequisite i) for admission to Australian Government accredited aged care homes, ii) to receive Community Aged Care Package(CACP) where a care coordinator will manage the complex care needs of the recipient, iii) to receive an extended Aged Care at Home(EACH) package where a similar range of care services as CACP is provided, iv) to receive an extended Aged Care at Home Dementia (EACH-D) package whereby the services is similar to EACH for people with Dementia; and v) for a place in a Transition Care Program (TCP) [40]. Most of these services are delivered by highly trained multidisciplinary care teams lead by 180 trained geriatricians and supported by the GPs throughout Australia [40].

### The way forward: fundamental solutions needed to bridge the gap

Increased longevity is not only a triumph for a society but a huge challenge for health systems. Developed countries have been facing challenges related to ageing for a long time and much is to be learnt from their experience. Mainstreaming ageing into global agendas is essential to prepare developing countries for the challenge. Although Malaysia is still considered a country with a relatively young population, its ageing population is steadily increasing. The cost of health care will increase due to the escalating burden of chronic illnesses in an ageing population. Proactive measures need to be taken to contain these costs.

These measures include restructuring the health care system. The recently announced proposal by the Malaysian government to set up a national health financing scheme which will integrate all public and private primary care services under a common network of care [42], offers promise to the vulnerable population. It is hoped that the implementation of this scheme will provide better coordination, continuity, equity and quality of care for older people and those with chronic conditions.

The 400 highly trained family medicine specialists are in unique positions to champion patient-centred and comprehensive primary care services under this new scheme. Focus should be shifted from providing care for self-limiting minor ailments to high quality preventive care, chronic disease management and aged care. These family medicine specialists must also take a leadership role in system redesign from a grassroots' perspectives. The numbers, however, are far from adequate as the projected number of family medicine specialists needed to deliver care to the population is 33,000 [43]. Therefore, specific measures are currently being undertaken by the public universities and the Academy of Family Physicians Malaysia (AFPM) to produce more family medicine specialists in order to meet the country's demand. The Diploma in Family Medicine (DFM) should be an important first tier qualification for all primary care providers. To become family medicine specialists, these clinicians should either possess the local Masters in Family Medicine, FRACGP/MAFPM or MRCGP (UK) qualifications, as currently recognised by the National Specialist Register for Family Medicine.

High quality training for primary care doctors is vital to ensure the delivery of quality chronic disease management and care of older people. The primary care curriculum, both at the undergraduate and postgraduate levels, should be revised to produce family medicine specialists who are not only clinically competent, but who can become effective gatekeepers, provide coordination and continuity for long term health problems; and lead a multidisciplinary primary care team. Malaysian medical schools should also incorporate geriatric medicine in their medical curricula to prepare future doctors to manage our ageing population in the years to come. Geriatric curriculum should also be included in the training of allied health personnel as there is a huge role for multidisciplinary team based care management of older people.

Availability of highly trained allied health personnel is critical to support the deployment of a multidisciplinary team in primary care. There must be a constant endeavour to increase the numbers of highly skilled nurses, pharmacists, dieticians, physiotherapists, occupational therapist, psychologists and other allied health personnel to deal with the challenge in managing chronic conditions in older people with complex health care needs. Assistance and training should be provided to the caregivers of older people in the community by this skilled team.

Health care workers must also ensure that older patients and their families have adequate information and skills to manage their health problems. The traditional role of patients as passive recipients of health care no longer holds true. Patient self management has been shown to reduce severity of symptoms [44] and was also found to be cost-effective [45]. Therefore, it is absolutely vital that patients are empowered with skills to manage their health, to enable them to work in partnership with their health care providers. This concept highlights a new paradigm in clinical practice and therefore requires effective communication and behavioural change skills of doctors and allied health personnel, which further underscores training needs in these areas for the primary care workforce.

Older persons are fully entitled to have access to comprehensive health care services which includes preventive care, curative care, chronic disease management and rehabilitative care. Preventive care for older people involves health promotion and disease prevention throughout life which focuses on maintaining independence and delaying the onset of disease. On the other hand, rehabilitative care involves improving the quality of life of older persons who already have disabilities. Some useful lessons can be learnt from the Australian health system in caring for their older people. These include conducting annual health checks for older population in primary care. There is also a need to set up community based aged care assessment teams to assess the elderly who may require special assistance since many of our elders are homebound. Comprehensive service packages for preventive care, self management support, chronic disease care and rehabilitative care for older people should be developed under the proposed healthcare restructuring funded by the national health financing scheme. The role of secondary care geriatricians and psycho geriatricians should also be expanded to provide better support to family medicine specialist on the ground to improve aged care services.

Development of efficient clinical information systems by using electronic medical records would ensure long term coordinated care of older people in the community [46]. In addition, integration of evidence based guidelines into patient care will be fundamental in translating evidence into practice [47].

Apart from improving health care services for older people there is a need to develop a collaborative network with community resources. There is a necessity to build more retirement villages; low level and high level care nursing homes and also a dire need to improve facilities for the disabled and transportation for older people in Malaysia.

## Conclusion

In conclusion, the challenges to care for older people in the Malaysian health systems are mounting. Major impediments include a shortage of specially trained physicians and allied health personnel in both secondary and primary care sectors, fragmentation of health care systems into public and private sectors; and absence of universal funding mechanisms to ensure accessible and equitable health care.

A comprehensive restructuring of the health care system funded by a universal mechanism is required for Malaysia to handle the rising tides of illness and disability associated with population ageing. The proposed national health financing scheme will offer a promise of care to this vulnerable population. Comprehensive benefit packages for older people must be made available under this new scheme. Measures to train the primary care workforce to rise up to the challenge of caring for long term health problems and an ageing population must be put in place. Older people and their carers must be empowered with knowledge, skills and attitudes to self manage. Effective use of electronic medical records and guidelines would facilitate coordinated care and improve outcomes. Ultimately, family medicine specialists must become the agents of change. They are ideally suited to lead multidisciplinary primary care teams and to work with various agencies to ensure better coordination, continuity and quality of care is delivered to older patients across time and settings.

## Competing interests

The authors declare that they have no competing interests.

## Authors' contributions

These authors contributed equally to this work. All authors have read and approved the final manuscript.
